# Syllabus of Gynæcology Based on the American Text Book of Gynæcology

**Published:** 1895-04

**Authors:** 


SYLLABUS OF GYNAECOLOGY BASED ON THE AMERICAN TEXT BOOK OF GYNAECOLOGY.—By J. W. Long, M. D., Richmond. Professor of Gynaecology and Pediatrics in the Medical College of Virginia. Gynaecologist to the Hospital of the Medical College of Virginia; Consulting Gynaecologist to the Richmond City Dispensary, etc., etc. Price $1.00. Philadelphia: W. B. Saunders, 925 Walnut Street. 1895.

The author states that this syllabus has a three-fold object: to be used as lecture notes, to enable the student to more intelligently follow and remember the lectures; and as a convenient reference for practitioners, the whole is based on the American Text Book of Gynaecology, believing that this work reflects the most advanced gynaecological thought and work.

The syllabus meets the first two objects well, for it will prove an aid to students in the lecture room and in "cramming" for the "green-room." The subject matter is conveniently arranged and the teaching correct as it is based on a book which ranks first in America as a text book of gynaecology. It will not be found useful for practitioners, who should take the time to consult books of larger scope when they have occasion to refresh their memories or to post themselves on subjects with which they are unfamiliar.

